# Must Interculturalists misrepresent multiculturalism?

**DOI:** 10.1186/s40878-017-0058-y

**Published:** 2017-09-08

**Authors:** Tariq Modood

**Affiliations:** Centre for the Study of Ethnicity and Citizenship, SPAIS, University of Bristol, 11 Priory Road, Bristol, BS8 1TU UK

## Abstract

Statements of and advocacy for interculturalism always seems to begin with a critique of multiculturalism and aspire to offer a new and alternative paradigm of diversity and citizenship. With particular reference to a recent publication, which marks the current state of the art debate between the two ‘isms’, I suggest that the critique is often not based on an engagement with multiculturalist authors but targets popular (mis)perceptions of multiculturalism. A consequence of this is that interculturalists fail to appreciate the limitations of their critique and of their claim to novelty. The newness of interculturalism may relate to the normative significance of the majority but less to intercultural dialogue or to an anti-essentialism. While interculturalism has a contribution to offer, eg, by a focus on micro-level interactions, on superdiversity and by challenging multiculturalists to think about the majority, it is best understood as a version of multiculturalism rather than as an alternative paradigm. Multiculturalism can benefit from the contribution of interculturalism but this may involve moderating interculturalist ideas so, for example, not abandoning an anti-essentialism that is consistent with the sociological reality of groups, or by taking on board the normative significance of the majority but without accepting the idea of a majority precedence. In this way what is of value in interculturalism can be taken on board within existing multiculturalist theoretical frameworks.

For over a decade there have been a stream of academic and policy publications introducing and promoting a pro-diversity strategy of integration called Interculturalism (IC). They joined a popular dissatisfaction with political Multiculturalism (MC) even to the extent of defining MC in terms of its demotic understanding and initially ignoring the political theory of MC.^1^ This disinterest has proved difficult to maintain as in recent years theorists of MC have begun to respond to the IC critique and the claim that IC is an intellectual improvement upon MC (beginning with Kymlicka, 2007 and Meer & Modood, 2012). Consequently, a debate has emerged in which I think each side has something to learn from the other and mutual provocation promises to improve the quality of each ‘ism’, including requiring IC to express itself as a political theory and not just in terms of policies and empirical studies in relation to say, education, the creative industries or urban governance. The collection of essays, *Multiculturalism and Interculturalism: Debating the Dividing Lines* (Meer, Modood & Zapata-Barrero, 2016) is to date perhaps the best expression of this new debate, a kind of ‘state of the art’ were it not that the debate is not yet so developed as to merit such a term.

The central claim of IC is that it is a new paradigm which is superior to MC and should replace it. It is argued that ‘multicultural policies are not fit for purpose’ and that ‘interculturalism is based upon an entirely different conceptual and policy framework and offers a new and progressive approach to how we learn to live with diversity’ (Cantle, 2016a, p. 133). For Zapata-Barrero, there is ‘a new paradigm that is taking shape in this second decade of the twenty-first century: intercultural citizenship’ (Zapata-Barrero, 2016, p. 53). Advocacy for IC not only always begins with a critique of MC but a dubious characterisation of MC, as has been observed by one of the non-IC leading critics of MC, who notes that ‘the intercultural alternative rests on a polemical view of multiculturalism that few of its advocates would endorse’ (Joppke, 2017, p. 37).

This is a theme I have dealt with a number of times (eg., Meer & Modood, 2012; Meer & Modood, 2013; Modood, 2014; Modood, 2016a; Modood, 2017 and Modood & Meer, 2012) and will here draw or build on these earlier works. This self-referencing arises out of a combination of defending my work and, alternatively, not over-repeating it and hence offering the reader the references to where more argument can be found. My argument has been that while there is not one version of IC – in fact there are at least two quite different versions, which are not compatible with each other (as Cantle 2016a, p.140 notes)^2^ – they do not offer an alternative political theoretical framework to MC but rather emphasise certain aspects of MC (while de-emphasising or rejecting altogether certain other aspects of MC), including ones that MCs have neglected, and so some aspects of each version is helpful to MC, and MCs should take on board. I have however, insisted that IC in most respects is not a new political theoretical framework as some of its key ideas were already part of MC as a political theory, sometimes foundational to it; and where they are new – to at least my version of MC – such as the idea of majority precedence/recognition, they are, suitably revised, compatible with MC and should be taken up by MCs. Whatever the political or rhetorical benefits in dropping the term ‘MC’ and repackaging its ideas as ‘IC’ (Kymlicka, 2016), it is intellectually mistaken to accept IC as new and superior to MC. The reason that ICs cannot see this is because they do not really engage with the political theory of MC – or even its policies (Modood, 2016a) – but with a stereotype of MC. Without repeating previous publications I would like to revisit this contention by looking in some depth at certain key ideas of MC in order to demonstrate that IC negative characterisations of MC may resonate with stereotypes and ‘common perceptions’ (Bouchard, 2016; Zapata-Barrero, 2016) but are not true of the political theory of MC. As I have been arguing for multiculturalism as a political idea, movement, discourse, a set of policies and a conception of Britishness for about thirty years a certain defensiveness will be evident here. As the raison d’etre and self-justification of IC is based on a critique of MC, a MC engagement with IC inevitably has to be reactive and defensive, and indeed, self-referential. My argument is two-fold: much of the intellectual content of IC consists of a critique of MC; and this critique ignores or misrepresents the published work of MCs like myself.

I will focus primarily on my own theorisation of MC, developed with particular reference to the debates and experiences of Britain and with particular reference to, and efforts to accommodate, British Asian Muslim political assertiveness and which generically I have called “multicultural citizenship”^3^ and specifically, “multicultural Britishness”.^4^ It is a form of multicultural nationalism centred on equal citizenship that cherishes historical identities—minority and majority—and embraces what some might call conservative institutions such as the House of Lords and state-funded faith schools, as well as new yearnings such as new hybrid identities or the celebration of St. George’s Day as a national day for England (Modood, 2010, pp. 113–115) but insists on their extension or adaptation to include ethno-religious groups and identities. I know my conception of multicultural nationalism owes much to several prominent authors (most notably Bhikhu Parekh and Charles Taylor), but perhaps it is not identical to any one of them and in any case cannot be held to be representative of political multiculturalism per se.^5^ I should add however that I do not see my position as one that only exists in theory. It is grounded in the British experience, which has progressively evolved since the 1960s and is centred on civil society activism and public debates after *The Satanic Verses* Affair to include Muslims in British public life (Stokke & Lybaek, 2016). It is best represented in policy terms in the report of the Commission on Multi-Ethnic Britain (2000, aka as the Parekh Report, after its chair) and in the actions of the first New Labour government of 1997—2001 (Modood, 2016a). The discussion here, however, will be mainly at a theoretical level and with relatively limited country-specific reference (though some at the end). The theory in question has thus a form of idealisation (as per Weber’s ‘ideal types’) drawn from a concrete experience and so fits that experience better than any other but to what extent it fits other cases is an open empirical question not discussed here.

While this paper is explicitly a response to certain IC claims and inaccuracies I do not wish it to be merely backward looking or merely defensive and so will offer a development of certain ideas. I do claim, however, that these developments are in the main based on ideas in the public engagement pieces in Modood (1992) (collecting items published during 1988–1992), the essays collected in Modood (2005) (originally published during 1996–2003) and Modood (2007/2013) and so *pace* Cantle are not ‘hasty revisionisms’ (Cantle, 2016a, p. 140).^6^ I note, however, that while my MC is a target of IC critique it may not be an unambiguous one. Firstly, some IC critique is explicitly not engaged with academic publications. Bouchard, for example, says that the MC he has in his sights is not the multiculturalism of theorists but of ‘the common perception’, namely, ‘the most widespread perception of multiculturalism currently in the West’ (Bouchard, 2016, pp. 90-91). On the other hand, I seem to be included as a target of critiques, especially by Cantle (2012b; 2013; 2016a and 2016b). Moreover, Nasar Meer and I are charged with hastily revising MC in the light of IC and pretending therefore that IC is only saying what MC has been saying all along: ‘claims that the new principles of interculturalism were ‘foundational’ to multiculturalism all along (Meer & Modood, 2012, p. 182) reflect this hasty revisionism’ (Cantle, 2016a, p. 140). Finally, the ambiguity I am referring to is compounded by the fact that some MC I endorse is also endorsed by Cantle and according to some authors I am quite close to a version of IC. Thus Cantle critiques the Quebecan IC, Bouchard thus: ‘Bouchard (2011) mirrors much of the reified, static and defensive form of identity management found in European forms of multiculturalism, whereas the Canadian government form of ‘multiculturalism’ is a little closer to the European idea of interculturalism’ (Cantle 2016a, p. 140). This is despite my beginning my 2007 book by offering Canadian government MC as an exemplar (Modood, 2007, pp. 16–17). At the same time Iacovino suggests that my understanding of (British) multiculturalism is closer to Quebecan IC than Canadian MC and closer also to Quebecan IC than Cantle’s IC is (Iacovino, 2016, p. 492). So, a more plural and complex conceptual map of MCs and ICs would have to be offered but I will not attempt to do that here, though the debate book that is my main reference point itself offers several rival conceptual maps (Meer, Modood, & Zapata-Barrero, 2016); see also Levey (2017).

Given all this one might wonder if there is anything that can be nailed down here as comprising the difference between MC and IC. The editors of the 2016 book, comprising of two committed to MC and one to IC suggest this list:

i) the status of dialogue, contact and interpersonal relations within respective approaches.
ii) the position of historical majority cultural forms – or majority precedence.
iii) The normative significance of recognising groups in addition to individual citzens.
iv) The status of minority religious communities and organisations.

I have on many occasions written about the centrality of ethno-religious minorities to contemporary multiculturalism and so I will leave that aside here and offer some thoughts on the other three as themes in the IC critique of MC.

## Dialogue as foundational to multiculturalism

Zapata-Barrero has argued that the core of intercultural citizenship is ‘essentially one basic idea: that the interaction among people from different diversity attributions matters, and that this has been overlooked by the multicultural citizenship paradigm, which has mainly concentrated on ensuring the cultural rights of diverse groups’ (Zapata-Barrero, 2016, p. 54). This indeed has been the central claim of IC since it began as a critique of MC. Positively, IC posits ‘the public sphere as a contact zone, and it is based on everyday personal experiences... concentrating on barriers to interaction’ (Zapata-Barrero, 2016, p. 56). Meer and Modood (2012) responded by arguing that this overlooks that dialogue is a foundational concept of MC, a suggestion rejected by Cantle (2016a).^7^ I continue to hold the view that dialogue in the context of identity-based tensions and the remaking of identity-based public sphere and citizenship has been at the heart of MC, so, let me explain the above point in some further depth.

To understand the foundational character of dialogue in multiculturalism, it is best to understand it as a deliberate contrast to the dominant way of doing Anglophone political theory in the last quarter of the twentieth century, in which John Rawls was the most important theorist. He looks to found politics and social justice on what free, rational individuals would collectively agree to after discussion. The discussants should be able to focus on what is good for individuals in general, or to put it differently, what all individuals would want after reflection, not only on what individuals like themselves would want. They must think selflessly—literally. Rawls designs a thought experiment, the centrepiece of which is what he calls “the Veil of Ignorance” (Rawls, 1971). For the deliberation of individuals to lead to the discovery of social justice or ‘fair terms of social cooperation’ (Rawls, 1985, p. 232) they must be made ignorant—stripped—of their specific identities such as their gender, their class, their nationality, culture, religion and so on. So none of the reasoners knows for example whether they are rich or poor, black or white, Christian or Muslim and so on. So no one will risk favouring laws and policies that unduly favour a particular class, race or religion in case when, at the end of the deliberation, the Veil is lifted and they (re)learn who they are, it turns out they are not of the group they favoured.

Rawls’ claim is, then, that the principles of social justice can only be worked out by individuals, intellectuals, law–makers, benign governments and so on to the extent that they approximate to being self–less or identity–less reasoners. That, however, means that dialogue among such individuals is not necessary because, stripped of all their differences, such reasoners are identical. One reasoner can in theory come up with the just solution without there having to be a dialogue among all the citizens. Moreover, behind the Veil of Ignorance, the debate makes no difference to what is valuable in the product of the debate. The product—the principles that a diverse society should live by—are not influenced by who is or is not included in the debate, and so they remain the same however the debate goes. That is to say, they are not influenced by the debate and could indeed have been known without any dialogue having taken place. More precisely, they are known by *reason* not by dialogue or by who participates in the dialogue.

Multiculturalists have rejected abstract reasoning by a sole reasoner or identical identity–less individuals in favour of dialogue. They assume that the context for politics is already thoroughly imbued with dominant ways of thinking and doing—with cultural orientations such as national history and language, with religious and/or secular perspectives, with institutional norms and so on—and that these contextual factors cannot be abstracted out so as to identify a set of principles of justice independent of cultural interpretations. Moreover, the relationship between the relevant parties is likely to involve domination–subordination or inclusion–exclusion and that the weaker or newer party is likely to lack recognition or be misrecognised (Taylor, 1994).^8^ Dialogue rather than identity–less reasoning will be relevant here for at least three reasons. Firstly, the solution to the problem, or the arriving of a principle by which to address the problem, needs an effort at cross–cultural understanding. It is not just a question of taking material interests into account, but a matter of (re) designing the shared public space and rules of conduct so diverse cultural commitments and needs are explicitly taken into account. So that the public space does not simply reflect the dominant culture, but is opened up to accommodate new or marginalised minorities. Secondly, this means that the solution is genuinely open. By this I do not mean that “anything goes”. Rather, that the solution cannot be predicted in advance in the way that, say, the final step of a piece of mathematical reasoning can, of which we say the answer was there all along waiting to be discovered. The dialogue makes a difference: it contributes to a growth of understanding that genuinely is novel or additional to what was present before and the quality or character of the dialogue is dependent on the participants. Not simply in terms of their power of reasoning but in terms of “where they are coming from”, so that with different parties a different outcome would have been reached. Thirdly, the dialogue is important not just in discovering an outcome, but in building a *relationship* of trust, co–operation and ultimately of belonging together between parties to the dialogue.

The multiculturalist political theorists I have in mind include Iris Young, who assisted people to understand themselves as oppressed and to discover themselves in collective identities such as black or gay and to thus develop a liberatory identity and group politics and using it to engage with other groups to institute a new form of democratic politics (Young, 1990). Charles Taylor’s idea of a dialogical ethics and politics based on “recognising” those whose distinct cultural identities have been dismissed, held in contempt or whose preservation is at stake—such as the identities of African–Americans in the USA or Francophone Quebecers in Canada—too is an example (Taylor, 1994). Interestingly, in his more recent work Taylor relates his approach to diversity to a Rawlsian idea, that of ‘an overlapping consensus’ (Rawls, 1987; Taylor, 2009).^9^ For Rawls, this referred to the body of laws and policies that those with different religious and cultural perspectives could all agree on by focusing on politics rather than their full set of religious and value commitments. Taylor rejects the idea of the identityless self (sometimes referred to as ‘the unencumbered self’) and abstract reasoning as the method for arriving at a consensus. He borrows and adapts ‘overlapping consensus’, but makes the process of arriving at it much more expansive and dynamic so that it in fact is best understood as consensus building; something not given but to be worked at, including through new interpretations of actors’ points of views, one of the things that one might expect from a dialogue (Taylor, 2009). James Tully has continually emphasised that cooperation under conditions of deep diversity or ‘multiplicity’ requires a ‘multilogue’ (Tully, 1995), and has proposed the idea of ‘public philosophy’, the questioning of society’s dominant assumptions in order to expose their contingency—their lack of necessity—and so open the way to identifying other possible ways of thinking and living (Tully, 2008).

Bhikhu Parekh explicitly makes intercultural dialogue central to his conception of multiculturalism (Parekh, 2000/2006). His interventions in relation to *The Satanic Verses* Affair, in which he argued against a freedom-of-speech absolutism and argued that angry Muslims must be given a sympathetic hearing, are exemplary (Parekh, 1989). While fully recognising that in such public controversies the majority dominate public discourse, and often in a manner that is not conducive to dialogue or mutual learning, he argues that multiculturalism is not about allowing each minority to-live-as-it-wishes relativism (Parekh, 2000/2006). Rather, it is about ensuring that there is a genuine dialogue and that the minority is allowed to express its point of view. While such dialogues inevitably have a majoritarian or status quo starting point, because even while wanting to express unfamiliar sensibilities and bring in new arguments, minorities are primarily trying to persuade the majority. This often takes the form of a minority arguing that what it is seeking is not so different to what the majority, at one time or another, has sought for itself. In so arguing the minority must justify itself by appealing to—even while seeking to modify—the existing ‘operative public values’ which structure public debate and what is thought to be legitimate or reasonable in that polity at the time (Parekh, 2000/2006, p. 267). I too have presented multiculturalism as a form of dialogical citizenship (Modood, 2007/2013, pp. 126–128; 116–118).

This, then, is what I mean by saying that intercultural dialogue is central to multiculturalism, even foundational to it. This is not a hasty revisionism. What is true, however, is that interculturalists have made their own distinctive addition; an emphasis on cultural encounters and everyday interaction in localities, schools, clubs, public spaces. As my example of the Rushdie Affair shows the importance of dialogue has been central to multiculturalism but has been mainly thought of at the level of public discourses and political controversies.^10^ Interculturalists have added the micro in terms of interpersonal cultural encounters and group dynamics at the level of youth clubs, neighbourhoods, towns and cities (Meer & Modood, 2012). Thus the interculturalists’ contribution to diversity theory and practice is to neglect the significance of macro-level dialogue, deny its presence as a multiculturalist [foundational] idea and to focus exclusively on the micro (Cantle, 2012a).^11^

## The reality of groups

For Cantle, it is not dialogue or the importance of interaction that is supposed to mark the superiority of IC over MC. Rather, more fundamentally, ‘the key difference between multiculturalism and interculturalism generaly revolves around the way in which personal and collective identities are conceptualised and instrumentalised.’ (Cantle 2016a, p. 140). In MC ‘communitities [are] encouraged to view their identities as special and fixed’ (p. 136) whereas IC recognises the present is characterised by superdiversity and by multiple and fluid identities (pp. 140–143). This explains why ‘Interculturalism reacts against the process of political ethnicisation of people, and against considering ethnicity a given notion in categorising people’ (Zapata-Barrero, 2016a, p. 61), stands against fixed categories and is able to allow hybridity to flourish (p. 59). Zapata-Barrero endorses Rogers Brubaker’s critique of MC (amongst others) as suffering from ‘groupism’, namely the ‘tendency to treat ethnic groups, nations and races as substantial entities to which interests and agency can be attributed’ (Brubaker, 2002, p. 164; Zapata-Barrero, 2016, pp. 61–62). Apparently, according to Cantle, Meer and I have belatedly come to accept this critique of MC: ‘The recent acceptance [ie., confession] of multiculturalism’s ‘groupist’ approach to identity (Meer & Modood, 2012) is to be welcomed, but the majority of academic and policy literature on multiculturalism is founded on the protection of ‘essentialised’, ascribed and static identity boundaries, in which these ‘pure’ forms are privileged’ (Cantle, 2016a, p. 142).

This is an old criticism in the subject area of equality and group identities, predating IC and has been extremely successful. By successful I mean that everybody has been persuaded, it is just about impossible to find anyone within the social sciences who does not agree that such identities are not based on cognitive or behavioural properties that are shared by all who may be members of a relevant group such as women, black people, gay and lesbians and so on. If, then, group members do not share a common essence then they cannot be simply demarcated from non-group members because there will be many cases where individuals are not simply on one side of the boundary or the other. So groups cannot have discrete, nor indeed, fixed boundaries as these boundaries may vary across time and place, across social contexts and will be the subject of social construction and social change. Since about the 1980s onwards these views have become increasingly widely accepted across the social sciences and I and most multiculturalists are part of this overwhelming consensus, which I will refer to by the term, ‘anti-essentialism’. Anti-essentialism is a powerful way of handling ascriptive discourses, of showing that various popular or dominant ideas about women, gays or Muslims are not true as such but are aspects of socially constructed images that have been made to stick on to those groups of people because the ascribers are more powerful than the ascribed. The latter, in their variety, are not reducible to the essentialistic generalisations and stereotypes that are characteristics of ascriptions but not of lived-in identities. Anti-essentialism, then, is an intellectually compelling idea, and a powerful resource in the cause of equality.

Anti-essentialism, however, can be variously interpreted. Let me briefly rehearse two interpretations which I discussed in my book, *Multiculturalism* (Modood, 2007/2013). The first is the sceptical interpretation that the critique kills the groups as real entities and they only live on as ascriptions or reactions to ascriptions or political make-believe. For example, Brubaker (2005) argues that ‘ethnicity, race and nation are not things in the world but perspectives on the world; ways of seeing, interpreting and representing the social world’ (p. 17). This scepticism seems to underlie Zapata-Barrero’s and Cantle’s endorsement of Brubaker’s critique of ‘groupism’ and ‘political ethnicisation’, the view that appeals to culture and ethnicity are not about things in the world but are perspectival impositions upon individuals who may wish to be free of them.

Sceptics do not necessarily want to kill off worthwhile political projects around say a black identity or feminism and so some allow for something called ‘strategic essentialism’ (Spivak, 1990; cf. Kristeva, 1981), where pretending that there is a black or national identity is permitted because of the politics but analysts know that these identities are only ‘necessary fictions’ (Hall, 1992, p. 254). Another way of doing it is that employed by Anne Phillips in her book, *Multiculturalism without Culture* (Phillips, 2007), the intellectual thrust of which, that cultural groups do not exist, simply transposes into accommodating groups when it comes to politics, without any sense of obligation to reconcile these divergent intellectual and political positions. I think groups are necessary to both social science and to anti-racism or egalitarian politics and so I offered an alternative interpretation of anti-essentialism. I suggested that Wittgenstein’s concept of family resemblance offers a way of recognising that just as it does not make sense to say that games or languages do not exist because they do not share a common, definitional essence, so the lack of group essences and discrete, bounded populations with unchanging characteristics was not a good reason to assert in an a priori way that groups did not exist (Modood, 2007/2013, chapter 5).^12^ Rather, we had to have a more flexible, looser and variable notion of a group and of group membership that allowed for open-textured and overlapping boundaries and overlapping memberships. If it seems difficult to reconcile this with our a priori concept of group, let us call the entities, ‘groupings’. The key point was that once we stopped demanding that groups measure up to our impossible definitions we would lose the temptation to conclude that groups suffered from an ontological deficiency, that they were merely ‘perspectives upon the world’, ontologically no superior to the products of Othering. The point is that Cantle and other interculturalists are mistaken in saying that multiculturalists assume that ethnic and religious minorities are discrete, bounded, static, homogeneous and reifed entities. Rather, this is an old tactic of misrepresenting an opponent’s views, critiquing the misrepresentation and concluding that the opponent’s views have been demolished.

Let us, however, return to the substantive question. Given, that the political theory and sociology of multiculturalism speaks of ethnic groups (not necessarily in a pure or singular form, but perhaps as or including the ethnoracial, ethnocultural, ethnoreligious and so on), what does ethnicity refer to? Ethnicity here refers to a group that is very likely to be subject to ascriptive ‘Othering’ yet which has an existence of its own and not just as the imagined Other of someone else. Namely that in relation to a group defined by descent there is an element of self-identification, and with it community norms and structures and the inter-subjectivity that constitutes a group or groupness. The question, then, becomes can this ethnicity be given any content? In my book, *Multicultural Politics*, I gave as illustration five dimensions of ethnic ‘difference’ that cannot be reduced to Othering or to external explanations, so let me draw and expand on those (Modood, 2005):

1. *Cultural Distinctiveness*: norms and practices such as arranged marriage, existence of specific gender roles or a religion. Of course these norms and practices will to some extent be contested within the group and will modify and perhaps even disappear over time. The cultural distinctiveness therefore does not merely lie in conformity but also in the fact that one feels one needs to engage with those practices. The way that some Muslim women are reinterpreting Islamic gender norms is a very good example.
2. *Disproportionality*: a group may be marked by a disproportional distribution of a characteristic that is not distinctive (eg., high unemployment); while the distribution may be a structural product of opportunities and obstacles within the wider society, it can shape attitudes within the group, as well as to the group, a sense that they are not typical but different (eg., poor, brainy, sporty etc). Moreover, the disproportional presence or absence of certain ethnic minorities in certain occupations may be to do with racism or features of a particular labour market but they may also be due to, for example, an ethnic group particularly favouring a certain profession (eg., medicine) or for example not working with meat products.
3. *Strategy*: responses to a common set of circumstances (eg., high unemployment) may lead some groups to become demotivated or politically militant or self-employed; where differential strategies persist they can come to contribute to group consciousness and to distinguish groups.
4. *Creativity*: some groups are identified with some innovations (eg., longer shop-opening hours, a clothes style or a musical genre) even though they get taken up by the mainstream.
5. *Identity*: membership of a group may carry affective meanings that may motivate or demotivate, eg., black pride in a history of resistance to oppression, eg., as Muslims we must aid fellow Muslims in a time of need.

All of these will be contingent on time and place, some may be true in a small number of time-place contexts, some may be more pervasive – these are empirical questions, part of what I called the ‘mode of being’ (as opposed to the ‘mode of oppression’), which is not a priori, transcendental, or asocial but is an object of multi-disciplinary, multi-methods social scientific inquiry. In which case to say that ethnic groups do not exist but are imposed by MC policies on heterogeneous populations is philosophically naïve, sociologically false and a wilful misrepresentation of multiculturalism.

As for the normative implications in relation to placing hybridity and fluidity over the existence and recognition of groups, I have insisted that we should not approach the question in the spirit of a binary. With minority ethnicities changing as a result of social processes, identity politics and individual choices (Modood, Beishon & Virdee, 1994), multiculturalism must embrace both hybridity and groups, while recognising that the difficult normative and political questions for liberal democracies are in relation to the accommodation of ethno-religious groups (eg., Modood, 1998). I will not repeat those discussions here but the next section discusses aspects of the normative significance of groups.

## The majority and multiculturalising national citizenship

Probably the most challenging IC idea for MC comes not from European versions of IC but from Quebec (which some European ICs are uncomfortable with, eg., Cantle, 2016a, p. 140). It is the idea of ‘majority precedence’, namely, that at some point or other, and without legal entrenchment, the rights of the majority may legitimately trump the rights of minorities. Gerard Bouchard has offered a direct and pithy statement of it in English (Bouchard, 2011). For him, the central feature of IC is that it frames the question of diversity in terms of a majority-minority duality, not the plurality characteristic of multiculturalism (Bouchard, 2011, pp. 441–445), and within this duality, there must be a clear, albeit non-legal, precedence. “While seeking an equitable interaction between continuity and diversity, interculturalism allows for the recognition of certain elements of ad hoc (or contextual) precedence for majority culture” (Bouchard, 2011, p. 451). This is challenging for multiculturalists because it would be fair to say multiculturalists of the kind being defended here have not explicitly addressed the issue about the majority (though see Levey, 2017). Multiculturalists have of course written much on the re-making of a national citizenship in order to make it more inclusive (Modood, 2007/2013, Uberoi, 2015a and 2015b), as that is central to multiculturalism as I understand it^13^; and MC—certainly my own work—assumes that minority accommodation is to take place in an ongoing historic nation-state. MC is not about opposing a given nation-state but of co-operatively and dialogically adapting it and re-imagining it, i.e., multiculturalizing it from the inside, ie., by citizens. So perhaps it is not entirely accurate to say that multiculturalists have neglected to note the normative significance of the majority *tout court*. Yet, on the whole, while the national culture is assumed as an appropriate normative context, multiculturalists do not explicitly discuss the concept of the majority or the idea of majority rights or recognition.^14^ In so far as multiculturalists distinguish between the majority culture and the public or civic culture, it is about the former’s tendency to dominate and pass itself off as the whole of the national culture. It is assumed that a minority culture can be identified as distinct from what it needs to be included into but much less is said about the majority culture in this respect. I confess to being guilty here. I have subsequently closely engaged with Bouchard’s argument for majority precedence and will not repeat that here but simply note that my conclusion was that a case can be made for acknowledging that the majority, no less than minorities, has a normative significance but not a special precedence (Modood, 2014).

Having admitted that multiculturalists like myself have not engaged much with the concept of he majority, I would like to do two things here. Firstly, I will state what I think has been the view of the concept of the majority implicit in MC. Secondly, I will go beyond that to develop further the concept of a national multiculturalism that I have advocated in the context of Britain.

What I have said to date about minorities in relation to the majority can be summarised in terms of two “protectionist” statements and two positive statements:

i) Protection from racism, cultural racism and Islamophobia (not from majority culture per se)
ii) There should be no insistence on assimilation but nor should there be any hinderance against uncoercive social processes of assimilation or self-chosen assimilation; different modes of integration should be equally welcomed
iii) There should be multicultural accommodation of minorities within shared public institutions
iv) Minorities should be able to make claims on the national culture and identity in their own ways and this remaking of national identity is part of multicultural citizenship and should be welcomed and encouraged by the majority.

So, while multiculturalists may need to think more about “the majority”, it is not the case that existing theories are *negative* about majority culture per se or even that multiculturalism is about protecting minorities from majority culture. Rather the above list allows us to note that one of the noticeable differences between MC and IC is the emphasis the former puts on anti-racism and beyond that to acknowledging the role of the political, of contestations and the challenging of power relations.^15^ Furthermore, one of the central arguments of MC, and implicit in the list, is that the majority culture already has recognition of some sort—that is what is meant by saying the liberal state is not neutral and does not have to be neutral—and so it is a matter of extending this valued condition to minorities. MCs like me clearly accept that liberal democratic states may promote a national culture (within liberal limits and respecting other group identities) and this would be of benefit to the society or polity as a whole. This is reflected in the importance I give to both British national identity and the role that the state may play in relation to it (Modood, 2007/2013, pp. 146–154/135–143). Moreover, it is not an ad hoc addition but follows from the core of my position which puts a special value on identity (Modood, 2007/2013, pp. 41–45/36–40). So, while Bouchard categorises MC as insisting on “no recognition of a majority culture” (Bouchard, 2011, pp. 441–442) I cannot be characterised as someone who considers appeals to majority cultural heritage as illegitimate per se. The multiculturalist point is that the predominance that the cultural majority enjoys in the shaping of the national culture, symbols and institutions should not be exercised in non-minority accommodating ways. So, the goal is legitimate but the constraints are not just about traditional liberal freedoms of the individual, which may be enough to ensure non-discrimination and non-coercive assimilation, but also respect for post-immigration ethnoracial, ethnocultural and ethnoreligious group identities. This respect is both a constraint on the kind of national cultural identity building that may be pursued but, more positively, it is an opportunity for creating a certain kind of national identity, namely one which includes those kinds of group identities in the revised or reformed national identity, critically reforming but without displacing the narrative of the majority within the national identity. Minorities may wish to contest dominant narratives which exclude them or fail to respect them and their contribution but they do not compete with the majority in a zero-sum game. I have argued that the process should be seen as a kind of egalitarian levelling up, where the minorities come to share the public space, not a form of dispossession of the majority. More positively still, that the accommodation of minorities should not be seen as a drag on the national identity but as a positive resource; not as diluting the national culture but vivifying and enrichening it. Whilst liberal nationalism is often offered in relation to facilitating the solidarity that enables social democratic redistribution of resources, the distinctive goal of multicultural nationalism is to allow people to hold, adapt, hyphenate, fuse and create identities important to them in the context of their being co-citizens and members of socio-cultural, ethnoracial and ethnoreligious groups.^16^

Levey has suggested that my acceptance of the cultural predominance of the majority is simply as an empirical fact and is not sufficiently normative (Levey, 2016, p. 211). Yet, Bouchard (2015, pp. 111–112), recalling that the Bouchard-Taylor report spoke of “de facto precedence” (Bouchard & Taylor, 2008, p. 214), himself abandons the term “majority precedence” as he believes that the idea he wants to state “simply reflects the historical demographic and sociological weight of the majority.” (Bouchard, 2015, p. 140). So, perhaps there is at least an evolving strand of Bouchard’s thinking that is converging on multiculturalism. In any case, let me give two examples to illustrate the general points that I have been making about a multiculturalist position in relation to national identity and the respective claims of the majority and the minorities. The examples illustrate two different but complementary, indeed mutually necessary, aspects of multiculturalism.

The Church of England is clearly an institutionalised feature of England’s and Britain’s historical identity. This is reflected in symbolic and substantive aspects of the constitution. For example, 26 Anglican bishops sit by virtue of that status in the upper house of the UK legislature, the House of Lords. It is the Archbishop of Canterbury that presides over the installation of a new head of state, namely the coronation of the monarch. Given the rapidity of changes that are affecting British national identity, and the way in which religion, sometimes in a divisive way, is making a political reappearance, I think it would be wise not to discard lightly this historic aspect of British identity, which continues to be of importance to many even when few attend Church of England services and when that Church may perhaps have been overtaken by Catholicism as the largest organised religion in the country. Yet, in my advocacy of a multiculturalized Britain I would like to see the Church of England share these constitutional privileges—which should perhaps be extended—with other faiths. However, multiculturalism here does not mean crude “parity”. My expectation is that even in the context of an explicit multifaithism the Church of England would enjoy a rightful precedence in the religious representation in the House of Lords and in the coronation of the monarch. This, however, would not be just a crude majoritarianism nor based only on its historical significance but also on its actual track record in furthering multiculturalism and its potential to play a leading role in the evolution of a multiculturalist national identity, state and society. Both the historical and the multiculturalist contributions to national identity have a presumptive quality, and usually they qualify each other, but where they are complementary the case for “establishment” is enhanced and most of all where there is simultaneously a process of inclusion of non-Anglican faith communities and humanists.^17^

My second example is about religion in non-denominational state schools. I think multiculturalism should support a compulsory religious education (RE) in which children of all faiths and none are taught about a variety of faith traditions and their past and current effects upon individuals and societies, upon the shaping of humanity, taught to classes comprising those of all religions and those of none. Such classes should certainly include the contribution of humanism as well as the atheistic critique of religion and can be combined with ethics as is the case in Quebec. In many countries there are advocates for RE as part of a national curriculum.^18^ The main issue in relation to majority precedence is in relation to religious instruction (RI), the induction into a specific faith. Broadly speaking there are two majoritarian possibilities. We have a society where there is a majority religion and that alone is allowed as RI, and minorities might be exempted from those classes but no alternative religious instruction is provided. Or secondly, the majority view is that there should be no RI in state schools, as in the USA or in France (except in state-funded religious schools). Is it fair to impose either of these policies on minorities that do want RI?

That is certainly an appropriate subject for a national dialogue but if after that certain minorities want RI as well as RE, then a truly national system, certainly a multicultural system, must make an effort to accommodate minority RI. In my understanding then, under both the majoritarian possibilities the minorities should have their religions instructed or worshipped within the national system. On the other hand, minorities do not have the right to stop the majority from including the instruction of their religion. We should not, for example, ask schools to cease Christian RI or worship or celebrating Christmas *because* of the presence of Muslims or Hindus; rather, we should extend the celebrations to include, for example, Eid and Diwali. Such separate classes and faith-specific worship needs to be balanced with an approach that brings all the children together and into dialogue; indeed, without that it would be potentially divisive of the school and of society. But where that is in place, voluntary pursuit of one’s own faith or philosophical tradition completes the multiculturalist approach to the place of religion in such schools. Learning together about different faiths, including what they have in common and being instructed in or inducted into one’s faith community heritage as a normal school occurrence and not something excluded from the school community are then the two mutually balancing aspects of multiculturalism.

These two brief examples, then, illustrate what I take to be a multiculturalist recognition of the legitimate claims and limits of majority culture. The idea of majority precedence, then, in the work of Bouchard and within Quebecan IC more generally is a significant difference from most accounts of MC. Despite it containing a rather simplistic account of MC that is not found in or even said to be found in the work of multiculturalists, the latter can benefit from engaging with it. Multiculturalists can accept the starting point of Bouchard’s arguments in question but not the conclusions. The arguments show that the majority culture has a normative significance, but not normative precedence if that means the majority is able to make normative claims that minorities are not, or that majority claims always trump minority claims, or that there is always some prima facie presumption in favour of the majority culture. Engaging with these arguments show that the kind of multicultural nationalism that I and Bhikhu Parekh have been developing (though see Uberoi, 2015a and 2015b for the suggestion that Parekh has a new and distinct understanding of national identity) have tended to take the majority for granted as a normative context without explicitly discussing it, yet there is nothing in that MC that stands in the way of doing the majority justice. Indeed, the opportunity to reflect on this theme makes me think that multicultural nationalism is well placed to balance the normative identity claims of the majority and the minorities that Quebecan ICs seek when they emphasise the importance of mutual recognition, reciprocity and balance, though they tend to go astray when they express this as “majority precedence”.

## Conclusion

Interculturalism seems to always begin with a critique of multiculturalism and aspires to be a new and alternative paradigm of diversity and citizenship. Too often the critique is not based on an engagement with multiculturalist authors but targets common (mis)perceptions of multiculturalism. A consequence of this is that interculturalists fail to appreciate the limitations of their critique and of their claim to novelty. The alleged new ideas of interculturalism may relate to the normative significance of the majority but less to intercultural dialogue or to an anti-essentialism. While interculturalism has a contribution to offer, eg, by a focus on micro-level interactions, on superdiversity and by challenging multiculturalists to think about the majority, it is best understood as a version of multiculturalism rather than as an alternative paradigm. This allows for an appreciation of micro-sociological studies without accepting the usual prefatory critique of multiculturalism found in such studies. My own version of multiculturalism can benefit from the contribution of interculturalism but this may involve moderating interculturalist ideas, for example, of not abandoning an anti-essentialism that is consistent with the sociological reality of groups, or by taking on board the normative significance of the majority without accepting the idea of a majority precedence. In this way what is of value in interculturalism can be taken on board within existing multiculturalist theoretical frameworks.

## Footnotes

1 I will also use MCs and ICs to refer to multiculturalists and interculturalists respectively.

2 Zapata-Barrero (2016) believes there are three different versions of interculturalism and which actually are three different complementary aspects of interculturalism. Alternatively, if we want to go beyond Europe and Quebec, we can also note *interculturalidad* in Latin America, which engages with the legacy of colonialism rather than immigrant settlement (Solano-Campos, 2016).

3 Of course Kymlicka had earlier described his own position in terms of multicultural citizenship, this being the title of his pioneering book (Kymlicka, 1995).

4 There is a major question whether such a citizenship based multiculturalism in countries such as Canada, Australia and Britain can be extended to cover ‘pendular migrant Polish plumbers, Chinese students who overstay, American high skilled workers (in the UK) etc’ (as one anonymous referee put it), not discussed here but extensively in Triandifyllidou in press (2017).

5 In recent years Taylor, despite being one of the best known political theorists of multiculturalism, has come to self-define as an interculturalist (Taylor, 2012).

6 Except, as I acknowledge later in the text, I have been stimulated by IC to think in new ways about the normative significance of the majority. The single short piece I would cite against the ‘hasty revisionism’ argument and as an indication of the underlying consistency in my understanding of multiculturalism is Modood (1998).

7 I had actually earlier argued that ‘far from simply supporting difference, the multicultural state may also need to encourage forms of social mixing and interaction, though this will be a two-way process and not just in relation to simply avoiding minority segregation’ (Modood, 2007/2013, p. 64/ p. 59).

8 An anonymous reviewer pointed out that Taylor’s recognition ‘is a one-sided relationship, a remedial act by the perpetrator toward the victim’. True, the recognition is remedial, but moving from misrecognition to recognition requires dialogue to understand the other as an other for itself and not just as an object. The effort to understand the other as having a life or consciousness besides its subordinate role comes from one side but requires an identity assertion communicated from the other side.

9 At this stage Rawls had moved away from the Veil of Ignorance methodology described earlier of his most famous work (Rawls, 1971).

10 Though Parekh (2000/2006), gives some attention to the quotidian in a multiculturally constituted common culture (pp. 219–224/ p. 168).

11 Loobuyck (2016, p. 230), too uses the macro-micro distinction to distinguish between IC and MC but he conflates it with a state—civil society distinction and does not see that MC is not confined to the state and extends to citizen-to-citizen relations (Modood, 2007/2013, chapter 6). Contrary to another distinction he makes, multiculturalism is not just about justice and neglects the importance of belonging: on the contrary, belonging is more central to multicultural nationalism as I understand it than either the liberal goal of furthering autonomy or the social democratic means of redistribution of resources.

12 Parekh (2000/2006) has his own way of avoiding essentialism in relation to the concept of culture (p. 144). See also the excellent Kymlicka (2015). Varun Uberoi has pointed out to me that Iris Young’s discussion of the concept of a group (Young, 1990, pp. 42–46) should be regarded as the earliest anti-essentialist discussion by a multiculturalist political theorist.

13 Will Kymlicka has written extensively on the re-making of a national citizenship but I hesitate to cite him here as I am mindful of Levey’s argument that Kymlicka is a liberal nationalist and therefore not a parity multiculturalist and therefore is not a good example of a multiculturalist that Bouchard is arguing against (Levey, 2016).

14 For a path-opening discussion and typology of the majority, see Orgad (2015). He, however, confines his normative discussion to uses of majority culture in relation to just two areas of policy, namely, immigration and naturalisation. This is partly because his interest in “majority rights” is only in those that can be expressed in law. MC is not only or even primarily focused on law and Bouchard, as stated above, is not arguing for majority rights in law, and neither MC nor IC is primarily interested in immigration and naturalisation but in state-citizen and citizen-to-citizen relations.

15 For a very good example see Commission on Multi-Ethnic Britain (CMEB, 2000).

16 A more fundamental difference between liberal nationalism and the multiculturalism I am defending is that the former usually places individual autonomy as the highest good but the latter is silent on the question of there being a single, highest good and operates at the level where a plurality of overlapping but also contested goods is seen as part of the condition of politics.

17 On the relationship between multiculturalism and moderate secularism, see Modood (2016b).

18 I appreciate that this will read oddly to US readers. They should bear in mind that the US is not typical of how religion is treated in schools in the western democracies. For example, all states of the European Union give funding either to religious schools or for religious education in state schools (Stepan, 2011, p. 117).
